# Mouse Basophils Reside in Extracellular Matrix-Enriched Bone Marrow Niches Which Control Their Motility

**DOI:** 10.1371/journal.pone.0070292

**Published:** 2013-09-27

**Authors:** Salete Smaniotto, Elke Schneider, Nicolas Goudin, Rachel Bricard-Rignault, François Machavoine, Mireille Dardenne, Michel Dy, Wilson Savino

**Affiliations:** 1 Laboratory of Cell Biology, Institute of Biological and Health Sciences, Federal University of Alagoas, Maceió, Brazil; 2 CNRS UMR-8147, Hôpital Necker, Université Paris Descartes, Paris, France; 3 Cell Imaging Platform, Hôpital Necker, Université Paris Descartes, Paris, France; 4 Laboratory on Thymus Research, Oswaldo Cruz Institute, Oswaldo Cruz Foundation, Rio de Janeiro, Brazil; University of Bergen, Norway

## Abstract

Basophils co-express FcεRIα and CD49b, the α-2 chain of integrin-type receptor VLA-2 (α2β1), which recognizes type-1 collagen as a major natural ligand. The physiological relevance of this integrin for interactions with extracellular bone marrow matrix remains unknown. Herein, we examined the expression of several receptors of this family by bone marrow-derived basophils sorted either *ex-vivo* or after culture with IL-3. Having established that both populations display CD49d, CD49e and CD49f (α-4, α-5 and α-6 integrins subunits, respectively), we addressed receptor functions by measuring migration, adhesion, proliferation and survival after interacting with matched natural ligands. Type I collagen, laminin and fibronectin promoted basophil migration/adhesion, the former being the most effective. None of these ligands affected basophil viability and expansion. Interactions between basophils and extracellular matrix are likely to play a role *in situ*, as supported by confocal 3D cell imaging of femoral bone marrow sections, which revealed basophils exclusively in type-1 collagen-enriched niches that contained likewise laminin and fibronectin. This is the first evidence for a structure/function relationship between basophils and extracellular matrix proteins inside the mouse bone marrow.

## Introduction

Basophils are rare, circulating cells that have long been ignored, because of the lack of reliable methods of identification. However, recent studies have provided more specific phenotypic and functional criteria revealing their non-redundant immunoregulatory functions in diverse immune responses and their potential importance as therapeutic targets to control allergic disorders [1]–[3].

Basophils derive from the bone marrow [4] where they differentiate before entering the blood stream and/or infiltrating inflammatory sites [5]. Despite abundant data on structural and functional properties of circulating basophils, including migration [6], virtually nothing is known about their interactions with the bone marrow microenvironment, before being released into the circulation. Such microenvironment is composed of fibroblast-like cells, osteoblasts and osteoclasts, which support survival, differentiation and proliferation of hematopoietic cells through secretion of soluble factors and extracellular matrix (ECM) proteins [7]. It remains unknown how basophils fit in this bone marrow architecture.

Herein, we compared *ex-vivo* with culture-derived basophils for their expression of integrins and evaluated their migration/adhesion in response to matching ECM ligands. Furthermore, using confocal microscopy on femoral bone marrow sections, we found that basophils reside in particular niches of the bone marrow, forming close contacts with type I collagen, laminin and fibronectin.

## Materials and Methods

### Ethics statement

All animal experiments were performed after approval by the Ethical Committee for Animal Experimentation of Paris Descartes University (officially agreed by the French National Committee for Animal Care and Use); being registered with the number P2.MD.163.10.

### Reagents

Murine recombinant IL-3 was purchased from R&D Systems (Abingdon, UK), whereas rat type I collagen, human cellular fibronectin, mouse laminin (laminin 111, derived from EHS tumor cells) and bovine serum albumin (BSA) were from Sigma Co. (St. Louis, USA).

CD49b^+^FcεRiα^+^CD117^−^ medullary basophils were analyzed for the expression of selected integrin α-chains CD49d, CD49e and CD49f, after staining with appropriate fluorochrome-conjugated mAbs from BD/Pharmingen (San Diego, CA). ECM architecture was visualized on normal mouse bone marrow acetone-fixed cryostat sections by immunofluorescence staining with unlabeled rabbit polyclonal antisera with specificities for mouse type I collagen, laminin or fibronectin (Novotec, St. Martin-La-Garenne, France), then revealed with rhodamine-coupled goat anti-rabbit IgG (Sigma). All antibodies are listed in table 1. Cell viability was estimated by XTT assays (Promega, Charbonnières Les Bains, France).

**Table 1:** Antibodies applied in cell enrichment, cytofluorometry, Immunofluorescence, cell migration and cell adhesion assays.

Fluorochrome-labeled primary monoclonal antibodies* (*anti-mouse proteins*)				
*Celular/Molecular specificity*	*Species origin and Ig isotype*	*Fluorochrome*	*Clone*	*Manufacturer*
NK1.1	Mouse IgG2a	−	PK136	StemCell Technologies
FcεR1	Hamster IgG1	FITC	MAR-1	eBiosciences
CD29	Hamster mAb IgG2a	Phycoerythrin	HM-β1–1	BD Pharmingen, San Diego, USA
CD49b	Rat mAb IgG1	Alexa 647	DX5	Biolegend,
CD49b	Rat mAb IgG1	APC	9F10	Biolegend,
CD49d	Mouse mAb IgG1	Phycoerythrin	R1–2	BD Pharmingen, San Diego, USA
CD49e	Mouse mAb IgG1	Phycoerythrin	5H10–27	BD Pharmingen, San Diego, USA
CD49f	Rat mAb IgG2a	Phycoerythrin	GoH3	BD Pharmingen, San Diego, USA
CD104	Rat mAb IgG2a	Phycoerythrin	346–11A	BD Pharmingen, San Diego, USA
CD117	Rat mAb IgG2a	Pacific Blue	2B8	BD Pharmingen, San Diego, USA
Purified unlabeled primary antibodies (*anti-mouse proteins*)				
Type I collagen	Rabbit polyclonal antibody	–	–	Novatec, St. Martin-La-Garenne, France
Laminin**	Rabbit polyclonal antibody	–	–	Novatec, St. Martin-La-Garenne, France
Fibronectin	Rabbit polyclonal antibody	–	–	Novatec, St. Martin-La-Garenne, France
Blocking monoclonal antibodies for cell migration and cell adhesion assays				
CD49b	Rat mAb IgG1	–	R1–2	BD Pharmingen, San Diego, USA
CD49d	Rat mAb IgG1	–	HMα2	BD Pharmingen, San Diego, USA
CD49e	Rat mAb IgG1	–	5H10–27	BD Pharmingen, San Diego, USA
CD49f	Rat mAb IgG1	–	GoH3	BD Pharmingen, San Diego, USA
Unrelated labeled antibodies				
Mouse IgG1	Goat polyclonal antibody	Alexa Fluor 594	–	Jackson Immunoresearch, Suffolk, UK
Mouse IgG2b	Goat polyclonal antibody	Alexa Fluor 488	–	Molecular Probes, Cergy Pontoise, France
Mouse IgG3	Goat polyclonal antibody	Biotinylated	–	Jackson Immunoresearch, Suffolk, UK
Rabbit Ig	Goat polyclonal antibody	Alexa Fluor 488	–	Jackson Immunoresearch, Suffolk, UK
Secondary antibodies				
Mouse IgG1	Goat polyclonal antibody	Alexa Fluor 594	–	Jackson Immunoresearch, Suffolk, UK
Mouse IgG2b	Goat polyclonal antibody	Alexa Fluor 488	–	Molecular Probes, Cergy-Pontoise, France
Mouse IgG3	Goat polyclonal antibody	Biotinylated	–	Jackson Immunoresearch, Suffolk, UK
Rabbit Ig	Goat polyclonal antibody	Alexa Fluor 488	–	Jackson Immunoresearch, Suffolk, UK

* MAb: monoclonal antibody; ^**^ This polyclonal antibody recognizes various laminin isoforms; FITC: fluoresceinisothyocyanate;

PE: phycoerythin.

### Mice

Male and female 6–8 week-old C57BL/6J mice were bred under pathogen-free conditions in our facility.

### Preparation of basophil-enriched cell populations

Basophils were prepared from freshly isolated or cultured bone marrow cells as reported before [8]. Briefly, bone marrow cells (BMCs) were flushed from mouse femurs. They were enriched for basophils by depleting NK1.1^+^ cells, followed by positive selection of CD49b^+^ cells using the Robosep automaton (Stem Cell Technologies, Vancouver, Canada). This procedure led to an enriched basophil population (around 40% of basophils), as defined by the FcεRIαCD49b^+^ phenotype. Alternatively, basophil-enriched populations were obtained by initial negative selection of CD117^−^ cells using the Robosep automaton, followed by phenotypic identification of FcεRIα cells.

*In vitro* basophil-enriched populations (around 90%) were also generated from total bone marrow cells cultured during 8–9 days in RPMI medium containing 10% FCS, 2 mM glutamine and 10 ng/ml IL-3 followed by positive magnetic sorting of CD49b^+^ cells with the same Robosep automaton as above.

### Immunofluorescence

Six μm thick femoral cryostat sections were assessed for simultaneous expression of CD49b, FcεRIα and one given ECM protein, as reported before [9], [10]. After nuclear staining with DAPI, samples were analyzed under a confocal fluorescence microscope (TCS SP5, Leica, Germany). Co-localization of two markers was defined after calculation of Pearson's coefficient with imageJ (v1.45) and JaCoP plugin, as described previously [11]. Volume profiles were built up using the *Imaris* software (v6.4.2, Bitplane Scientific Software, Zurich, Switzerland), after analyzing stacked confocal images. The occupancy of ECM lattices by basophils was quantified by measuring the volume filled by basophils (phenotypically defined by co-localization of CD49b and FcεRIα staining) over the volume of a particular ECM network, considering an average thickness of 6 µm for each cryostatic section. At least 35 confocal slices, from 6 to 12 microscopic fields were screened. The same procedure was applied with respect to type I collagen, fibronectin and laminin.

### Real Time PCR

Total RNA was isolated from basophil-enriched population (95–98% of cells bearing the phenotype FcεRIαCD49b^+^) derived from IL-3-induced bone marrow cell cultures by using the RNeasy Micro Kit (Qiagen, Courtaboeuf, France) according to the manufacturer's instructions. Total RNA concentration and purity were determined using a Nanodrop ND8000 spectrophotometer (Thermo Scientific NanoDrop Products, DE, EUA). Reverse transcription were performed using RT^2^ First Strand Kit (SA Biosciences, Courtaboeuf, France). Screening of expression for integrin-type receptors was performed by using Mouse Extracellular Matrix & Adhesion Molecules PCR Array (SA Biosciences, Courtaboeuf, France), based on SYBR Green Quantitative real-time PCR technique, and carried out according to the supplier's instructions. The expression of the following genes were ascertained: ITGA2, ITGA3, ITGA4, ITGA5, ITGAE, ITGAL ITGAM, ITGAV, ITGAX, ITGB1, ITGB2, ITGB3 and ITGB4.

Endogenous controls used in the normalization between the different amplified samples were selected among ACTB, GAPDH and HPRT1 genes by the method geNorm [12]. Amplification data were analyzed using a R statistical package, version 2.14.0 [13]. Two independent samples were analyzed in triplicates. Although we carried out two independent experiments for detecting integrin chain transcripts, using in both cases the qPCR strategy, we only applied one condition of basophil enrichment, namely cell sorting of IL-3-induced bone marrow cell cultures. Since results were quite clear-cut, in terms of transcript amplification as well as lack of gene expression, in the results section we have listed the ITGA and ITGB genes that were assayed, stating those in which transcripts were clearly detected.

Assay for detection of gene expression for integrin alpha 11 was performed separately, by a SYBR Green qPCR using a QuantiTect Primer Assay (Qiagen, Courtaboeuf, France) according to the manufacturer's instructions.

### Cytofluorometry and cell migration/adhesion assays

Following the 4-color cytofluorometric staining, basophil-enriched bone marrow cells were acquired in a FACScanto® cytometer (Becton Dickinson, San Diego, USA) and analysed with FloJo software as reported before [14].

Migration or adhesion assays were performed as reported for lymphocyte populations [15]–[17]. In brief, 5-μm pore size transwell plates or 24-well culture plates, respectively, were coated with 10 μg/ml mouse type I collagen, fibronectin, laminin or bovine serum albumin (BSA, negative control) for 1 h at 37°C. Basophils (2×10^5^ cells) were then plated into the upper chambers or allowed to adhere on the pre-coated plates. After a 3-h or 1-h incubation, respectively, cells that had migrated or adhered were removed, counted and analyzed by flow cytometry as described above. The % input corresponds to the percentage of FcεRIα^?^CD49b^+^ cells within the total population of this phenotype having migrated in response to ECM proteins, minus the percentage corresponding to the BSA control. In additional experiments, basophils were enriched as FcεRIαCD117^−^ cells.

Total numbers of adhered basophils were calculated after subtraction of background adhesion to BSA. Specificities of migration and adhesion were ascertained by blocking experiments with matching integrin-type ECM receptor neutralizing monoclonal antibodies.

### Evaluation of basophil survival and proliferation

We determined how interactions with integrin ligands affected survival and/or proliferation/differentiation of the basophil lineage. We applied the XTT assays to evaluate survival of purified mature basophils selected either positively by the expression of CD49b or negatively by depleting CD117^+^ cells, from IL-3-stimulated 8-day cultures of bone marrow cells. In both cases, cells were led to interact for 24 h with ECM-coated plates (10 μg/mL) of type I collagen, fibronectin, laminin or the three ligands together. XTT was then added and absorbance was measured using a Versamax ELISA microplate reader (Molecular Devices, Sunnyvale, CA), as recommended by the manufacturer.

In a second vein, we looked for the possible effect of ECM ligands on proliferation and/or differentiation of the basophil lineage by determining the relative basophil (CD49b^+^FcεRIα^+^ cells) counts generated from bone marrow cells cultured for 8 days with IL-3 with or without ECM ligands (10 μg/mL).

## Results and Discussion

### Medullary basophils express functional integrin-type ECM receptors

**Figure 1:**
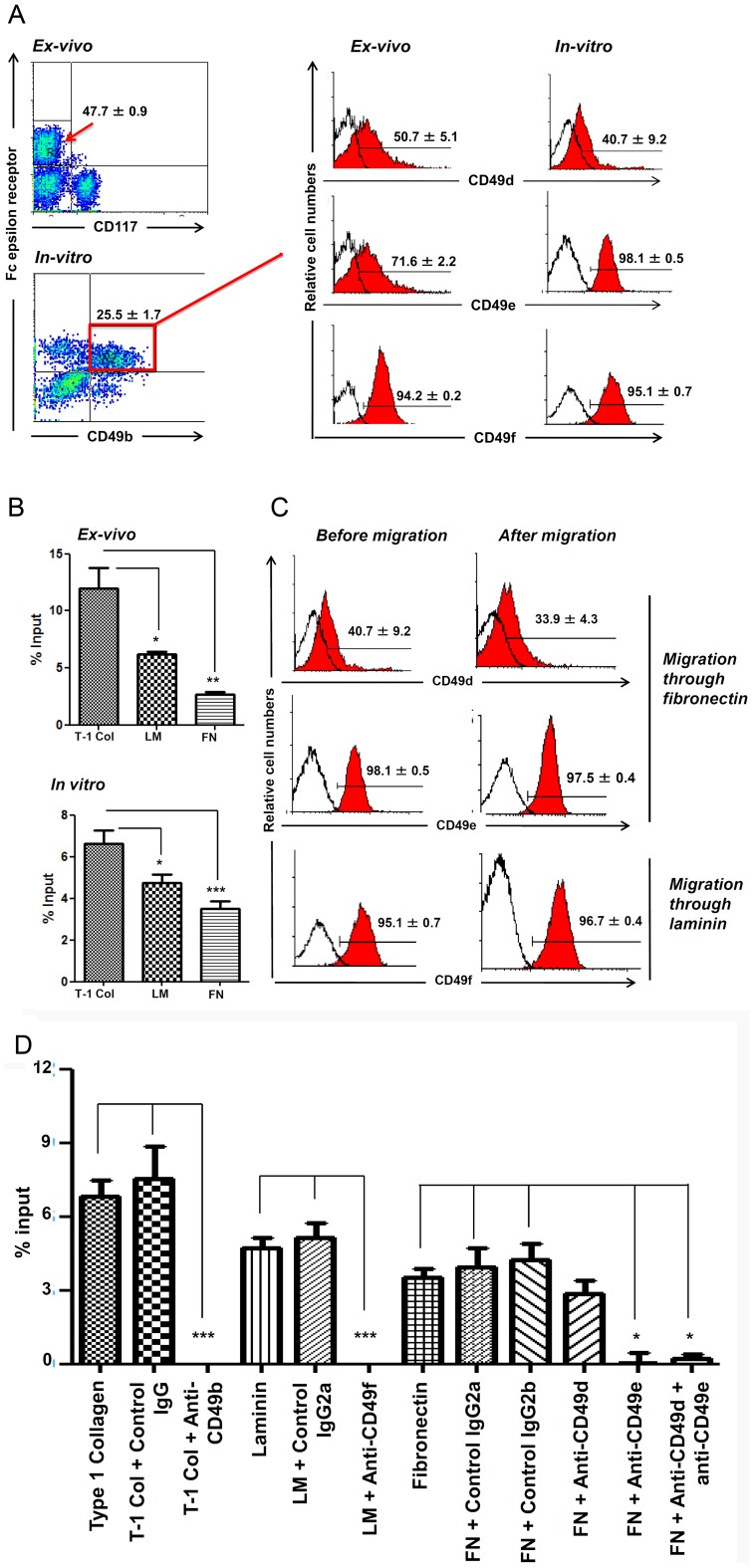
Bone marrow-derived basophils express integrin and migrate in response to their respective extracellular matrix ligands. The procedure used for direct isolation of resident medullary basophils yields a population that contains 30–50% CD49b^+^FcεRIα^+^ basophils [8]. Basophil-enriched populations were also generated from total bone marrow cells cultured for 8 days with IL-3, as described before [8], [9]. Panel **a** depicts cytofluorometric profiles of constitutively expressed integrin-type ECM receptors for type-1 collagen, fibronectin and laminin, on basophils isolated ex vivo or from IL-3-driven cultures. Panel **b** shows that freshly isolated (*ex vivo*) or bone marrow-derived basophils (*in vitro*) migrate through collagen, fibronectin and laminin, and that the migratory response is highest in response to type-1 collagen, as compared to laminin and fibronectin. Panel **c** reveals that in all cases, membrane expression of corresponding receptors is not changed. Panel **d** shows that neutralizing monoclonal antibodies to CD49b, CD49f and CD49e completely abrogated specific migration through type-1 collagen, laminin and fibronectin, respectively. Experiments in this figure were performed with 2–3-month-old mice, with at least 5 animals being evaluated *per* group. In all panels, values correspond to specific migration after subtracting background adhesion to wells coated with BSA. Results are expressed as means ± SE. * *p*<0.05; ** *p*<0.01; *** *p*<0.001.

We first used flow cytometry analysis to determine in bone marrow basophils, the expression of selected alpha-integrin chains, namely, CD49b, CD49d, CD49e and CD49f, which form with the β1 subunit, the VLA (very late antigens) ECM receptors VLA-2 (α2β1), VLA-4 (α4β1), VLA-5 (α5β1) and VLA-6 (α6β1), respectively. These integrins were all constitutively expressed, whether basophils were sorted *ex-vivo* or expanded in cultures set up with IL-3 (Fig. 1a).

Gene expression analysis of integrin α or β chains, revealed that basophils actually contain transcripts for a variety of integrin α and β chains, namely ITGA2, ITGA4, ITGA5, ITGAL ITGAM, ITGAV, ITGAX, ITGB1, ITGB2 ITGB3, whereas transcripts for ITGA3, ITGAE and ITGB4 as well as the ITGA11 gene, were not amplified, Considering these data, we further assessed, by cytofluorometry on basophil sorted population, the membrane protein expression of two selected integrin chains: CD29 (the β1 integrin chain, encoded by the ITGB1 gene, and the forms the VLA integrins, including VLA-2, VLA-4, VLA-5 and VLA-6) and CD104 (the β4 integrin chain, encoded by the ITGB4 gene). These data are summarized in figure S1. As expected, CD29 was largely expressed by basophils whereas no specific fluorescent signal was seen when we attempted to detect CD104. In both cases, results coincided with the transcript data obtained in qPCR experiments.

Functionally, we assessed migratory responses to the respective ECM proteins and found that basophils, from either preparation, did migrate in *transwell* chambers through the molecular lattice of all three matching integrin ligands; type I collagen being more effective than fibronectin or laminin (Fig. 1b). Note that a certain amount of nonspecific migration through BSA occurred in these conditions. Hence, the values given in figure 1b and d represent the specific migration, after subtraction of the number of cells having migrated through BSA.

The cell surface expression of integrins was not modified by exposure to their ECM components (Fig. 1c). Consistent with a specific effect, ECM-driven migration of culture-derived basophils was blocked by monoclonal antibodies against integrin α-chains CD49b, CD49e and CD49f (Fig. 1d).

Mirroring the effect on the migratory response, a certain number of basophils adhered to a BSA coat. However, as shown in figure S2, adhesion to the type I collagen lattice was clearly much stronger, whether cells were selected positively by CD49d expression or negatively by the lack of CD117.

**Figure 2:**
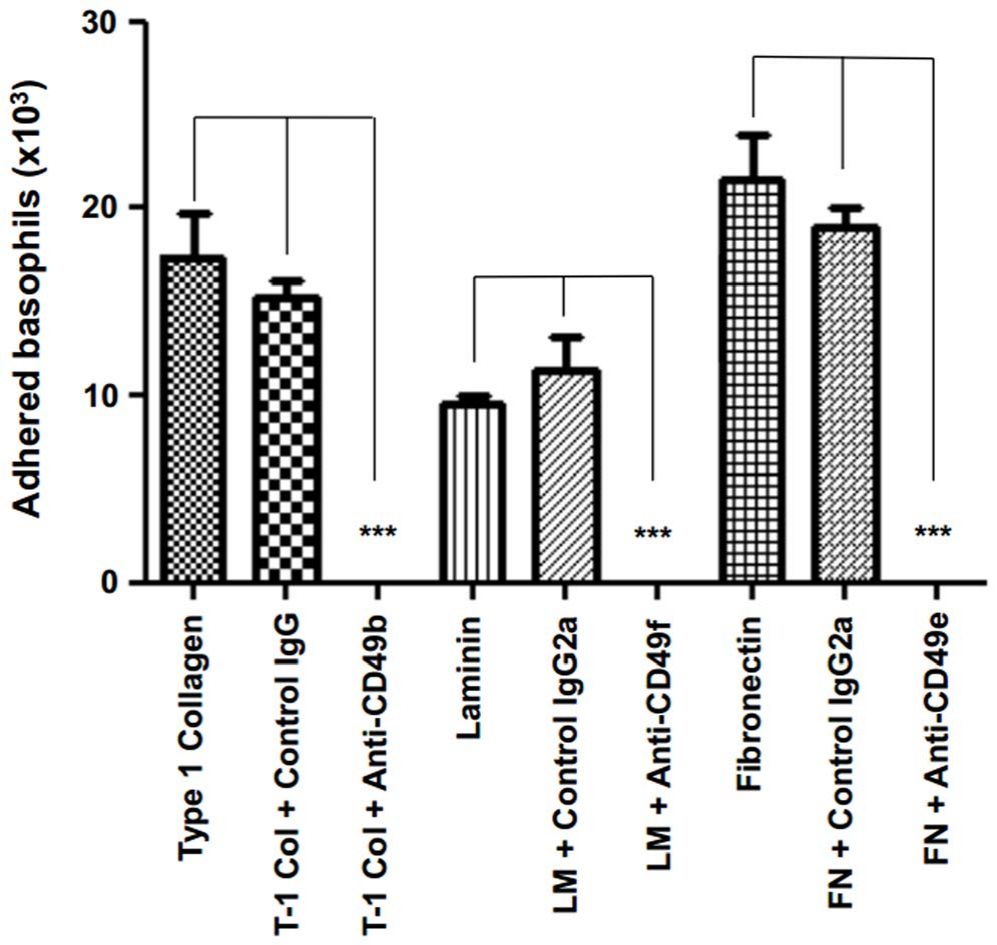
Bone marrow-derived basophils adhere onto purified extracellular matrix through integrin-type receptors. The panel shows that basophils obtained from IL-3 stimulated bone marrow cultures are able to adhere onto selected ECM proteins, namely type-1 collagen, laminin and fibronectin. Freshly isolated basophils behave similarly. Adhesion is abrogated by monoclonal antibodies that recognize matching integrin-type receptors. Experiments were performed with 2–3-month-old mice, with at least 5 animals being evaluated *per* group, and the adhesion assay was performed in 24-well culture plates coated with ECM proteins or BSA. Total numbers of adhered basophils correspond to specific adhesion after subtracting the background values from plates coated with BSA. Results are expressed as mean ± SE*** *p*<0.001.

As in cell migration assays, basophil adherence to purified type I collagen, fibronectin and laminin was specific since it decreased upon addition of the matching anti-integrin antibodies (Fig. 2).

**Figure 3:**
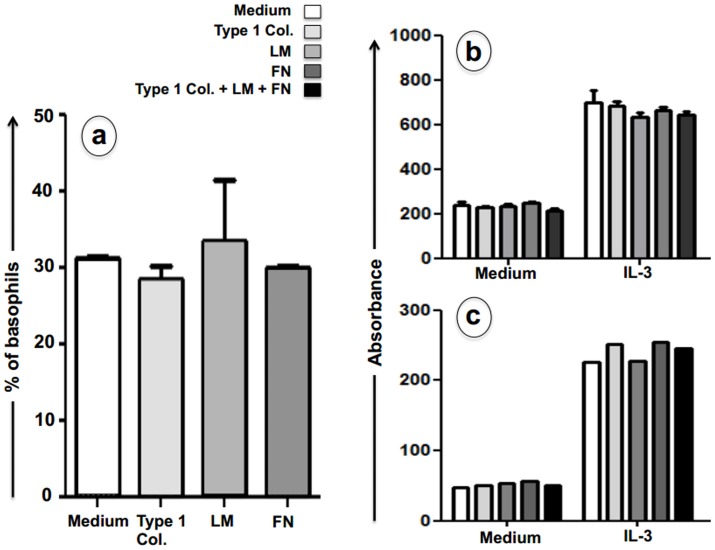
Extracellular matrix ligands do not modulate expansion or survival of basophils derived from bone marrow cultures. Percentages of basophils (phenotypically defined by CD49b-FcεRIα co-expression) expanded from bone marrow cells cultured with IL-3 remain unchanged whatever the ECM ligand added during culture: type I collagen (T-I col), fibronectin (FN) or laminin (LM) (panel **a)**. Lack of ECM ligand effect on survival of mature basophils as assessed by means of XTT staining of viable cells, quantified by absorbance (panels **b** and **c**). Results represented in panel b were obtained by positive selection based on CD49b expression and confirmed with one experiment using depletion of CD117^+^ cells (panel **c**). Cells were incubated during 24 hours in culture medium alone or supplemented with IL-3 and no significant differences were induced by the addition of integrin ligands. Data are expressed as means ± SE from three distinct experiments except for Fig. 3c that represents one experiment.

The proliferation and/or differentiation of the basophil lineage during culture of bone marrow cells with IL-3 was not altered in the presence of type I collagen, fibronectin, laminin during all the culture (Fig. 3a), In addition as shown in Fig. 3b–c, neither one of the three integrin ligands alone or all three together modified the viability of already sorted mature basophils cultured 24 h with or without IL-3. Taken together, these data show that the ECM interacts with bone marrow-derived basophils to modulate their adhesive and migratory properties, without affecting their proliferation and/or survival.

### Basophils reside in extracellular matrix-enriched bone marrow microenvironments

**Figure 4:**
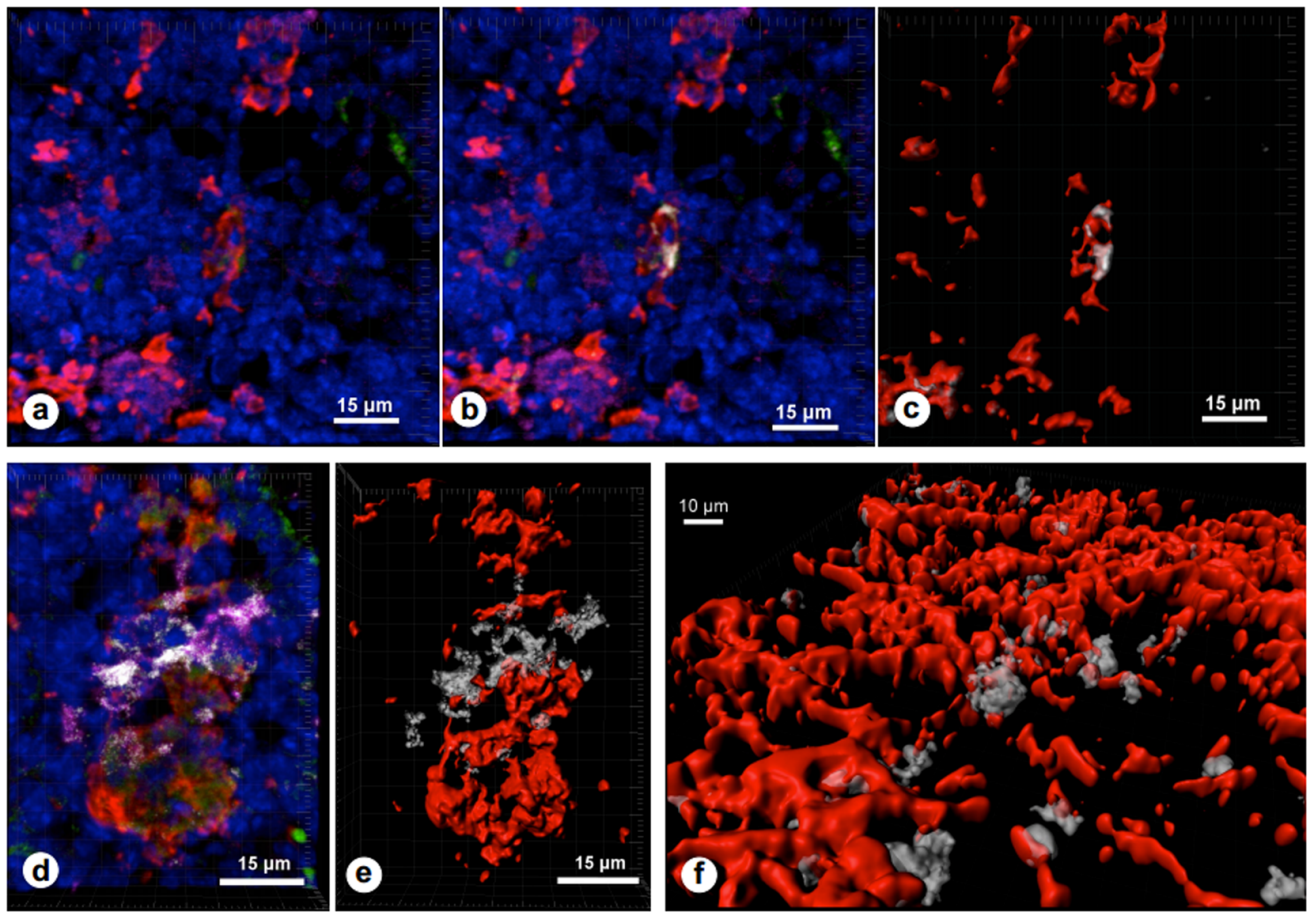
Basophils reside in extracellular matrix-enriched bone marrow microenvironments and adhere onto ECM ligands. Panel **a** represents a confocal image in which cell nuclei are labeled in blue by DAPI, type I collagen is seen in red, whereas the two basophil markers CD49b and FcεR1 are in green and magenta, respectively. Basophils were defined, using of Pearson's coefficient to identify CD49b and FcεR1 co-localization, and are shown in white in panel **b**, whereas the corresponding volume profile can be seen in panel **c**, where one basophil is seen in close contact with type I collagen fibrils. Panel **d** shows another microscopic field, in which a cluster of basophils can be identified (in white), in close apposition to fibronectin, thus showing that the basophil niche also contains fibronectin (labeled in red). The volume reconstruction of this same field can be seen in panel **e**. Panel **f** shows volume profiles in which it becomes clear that basophils (seen in white (defined by CD49b plus FcεR1 co-localization) are anchored in the fibronectin network (in red). Co-localization of two markers was defined after calculation of Pearson's coefficient with imageJ (v1.45) and JaCoP plugin, whereas volume profiles were settled using the *Imaris* software, after analyzing stacked confocal images. In all pictures, corresponding magnifications are shown in the white bars.

We postulated that ECM receptors and matching ligands might play a role in basophil distribution and motility within the bone marrow. Hence, we analyzed the structural relationship between basophils and ECM, using immunofluorescence to define the co-localization of basophils with type I collagen, laminin or fibronectin *in situ* on frozen sections of femurs from newly weaned mice. The rare basophils identified as FcεRIα^+^CD49b^+^ cells, were scattered among huge numbers of hematopoietic cells, whose nuclei were easily visualized by DAPI staining (Fig. 4a–c). Their diameter, determined for 20 cells, ranged from 10 to 15 μm, in accordance with the values reported [18].

Basophils were exclusively located in well-defined niches enriched in type I collagen, where they established close contact, clearly revealed by computer-based 3-dimensional reconstruction of stacked confocal sections further featured for volume profiles (Fig. 4c). In some microscopic fields, basophil cytoplasmic processes were intermingled with the type I collagen lattice. This pattern was seen, whether basophils were isolated or occurred in clusters. Basophils were not found in type I collagen-free regions of the hematopoietic microenvironment. Each of these niches also contained laminin and fibronectin (Fig. 4d–f), which were more uniformly distributed among bone marrow cells. Quantitative analyses of the relative volume of basophils in each ECM lattice revealed that these cells occupy around 15.6% of the volume occupied by type I collagen fibrils; 9.2% of the laminin lattice and 4.6% of the fibronectin network.

The importance of a suitable cell matrix for the growth of hematopoietic cell lineages has been acknowledged for many years. Matrix proteins participate in cellular attachment, migration and activation, tissue growth and repair, survival and differentiation [19]–[21]. However, because the surrounding bone made the cutting of sufficiently thin sections difficult, the 3-dimensional architecture of the bone marrow *in situ* has only just begun to be explored. Structural analysis has been used to characterize the stem cell niche [22] and the localization of several myeloid and lymphoid populations [23]. Integrin expression by basophils and interactions with ECM proteins have barely been investigated, apart from a few studies in human cells dealing with recruitment to sites of inflammation [24], [25], and information is still lacking concerning bone marrow basophils in mice deficient for a given integrin chain.

Our findings indicate that basophils reside in a 3D composite extracellular matrix, in support of the notion that their mobilization from the bone marrow might be triggered, not only by type I collagen, but also by other ECM ligands, as for example fibronectin and laminin.

To our knowledge, this is the first evidence for a structure/function relationship between basophils and ECM proteins inside the intact mouse bone marrow. Although colocalization of cells and type I collagen (or fibronectin and laminin) does not necessarily mean that these proteins form essential components of the basophil niche and the cells attach to them *in vivo*, the evidence provided herein does indicate that this may happen, since basophils are seen in close contact with and specifically adhere to ECM lattices.

Lastly, our data may be helpful for future explorations of basophil behavior within the bone marrow microenvironment in health and disease, in particular during anti-allergic therapies.

## Supporting Information

Figure S1 **Membrane expression of CD29, but not of CD104 in mouse medullary basophils.** The cytofluorometric profiles shown in this figure clearly demonstrate that mouse basophils express large amounts of CD29, the integrin β1 chain, whereas no membrane expression was seen for CD104, the integrin β4 chain. Red curves correspond to the use of isotype-matched unrelated Ig, couple with the same fluorochrome. The cells used in these assays were isolated from sorted FcεRIαCD49b^+^ basophils (>98% pure) derived from bone marrow cells cultured for 8 days with IL-3.
(TIFF)

Figure S2 **Adhesion of basophils to type I collagen lattice.** Total number of basophils adhering to a BSA or type-1 collagen coat after a 1-hour interaction. Cells were tested without enrichment (total), after positive selection of CD49b^+^ cells or depletion of CD117^+^ cells. In all cases, bone marrow-derived cells were generated during 8 days of culture in the presence of IL-3. Independently from the mode of selection, adhesion to type-1 collagen (type-1 col) was consistently higher than the values corresponding to nonspecific adhesion onto BSA. Adhered basophils were defined phenotypically by the co-expression of CD49b plus FcεR1α. Data were expressed as means ± SE with n = 3 for each group. ** p<0.001.
(TIFF)
