# Supplementary figures and images for: Mouse Basophils Reside in Extracellular Matrix-Enriched Bone Marrow Niches Which Control Their Motility
Source: PLoS One. 2013 Sep 27;8(9):e70292. doi: 10.1371/journal.pone.0070292 (PMC3785469; doi:10.1371/journal.pone.0070292)

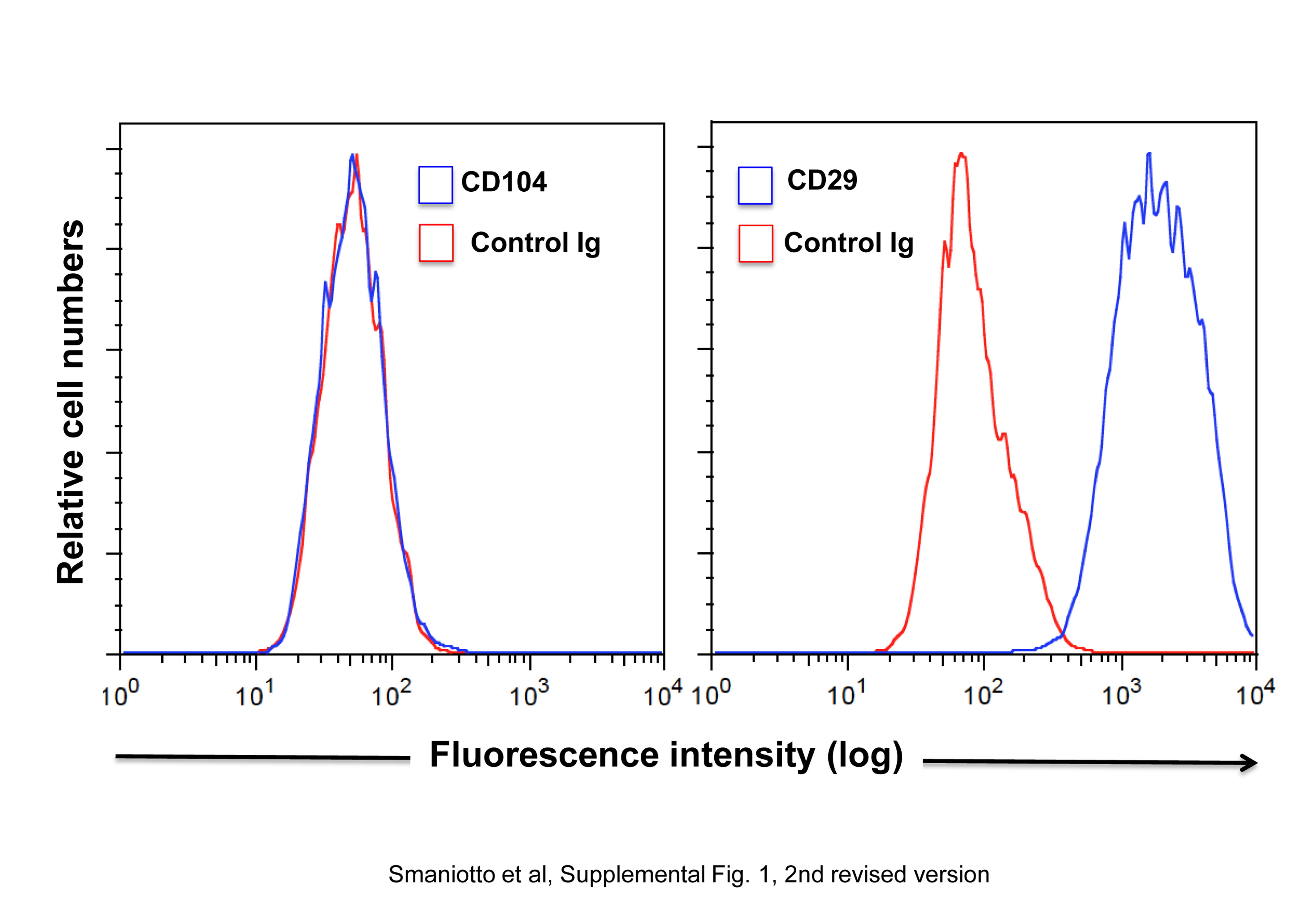

Supplement: Figure S1 — Membrane expression of CD29, but not of CD104 in mouse medullary basophils. The cytofluorometric profiles shown in this figure clearly demonstrate that mouse basophils express large amounts of CD29, the integrin β1 chain, whereas no membrane expression was seen for CD104, the integrin β4 chain. Red curves correspond to the use of isotype-matched unrelated Ig, couple with the same fluorochrome. The cells used in these assays were isolated from sorted FcεRIαCD49b+ basophils (>98% pure) derived from bone marrow cells cultured for 8 days with IL-3. (TIFF) [file pone.0070292.s001.tiff]

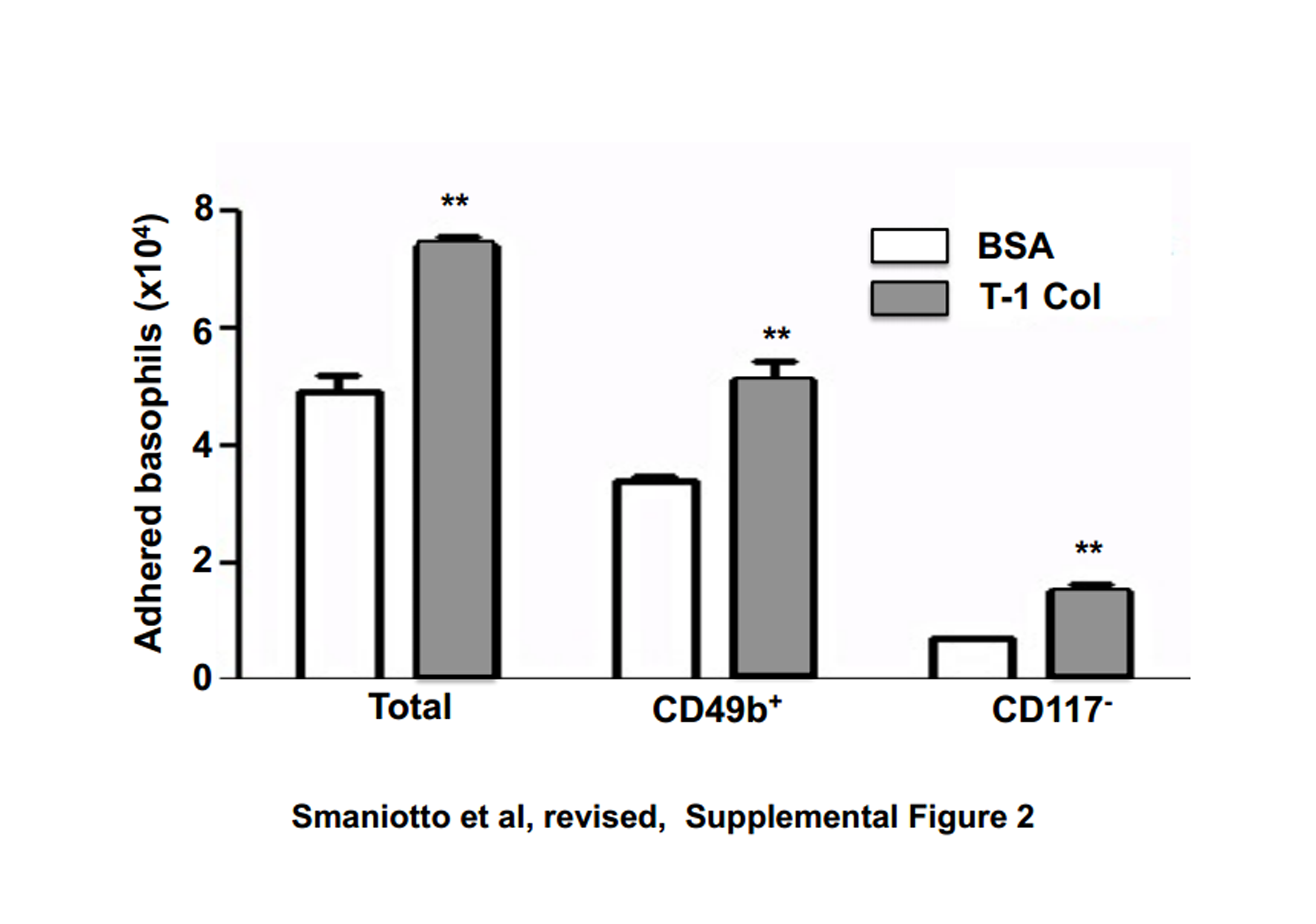

Supplement: Figure S2 — Adhesion of basophils to type I collagen lattice. Total number of basophils adhering to a BSA or type-1 collagen coat after a 1-hour interaction. Cells were tested without enrichment (total), after positive selection of CD49b+ cells or depletion of CD117+ cells. In all cases, bone marrow-derived cells were generated during 8 days of culture in the presence of IL-3. Independently from the mode of selection, adhesion to type-1 collagen (type-1 col) was consistently higher than the values corresponding to nonspecific adhesion onto BSA. Adhered basophils were defined phenotypically by the co-expression of CD49b plus FcεR1α. Data were expressed as means ± SE with n = 3 for each group. ** p<0.001. (TIFF) [file pone.0070292.s002.tiff]
